# Advancing Antimicrobial Stewardship With Diagnostic Excellence: The Role of Bayesian Reasoning

**DOI:** 10.1093/ofid/ofag213

**Published:** 2026-04-23

**Authors:** Ephraim L Tsalik, Neha K Prasad, Lee A Fleisher

**Affiliations:** Adjunct Associate Professor of Medicine, Duke University School of Medicine, Durham, North Carolina, USA; Emergency Department, Durham VA Health Care System, Durham, North Carolina, USA; Danaher, Washington, District of Columbia, USA; Rubrum Advising, Bala Cynwyd, Pennsylvania, USA; Rubrum Advising, Bala Cynwyd, Pennsylvania, USA; Professor Emeritus of Anesthesiology, University of Pennsylvania, Rubrum Advising, Philadelphia, Pennsylvania, USA

**Keywords:** bacterial vaginosis, Bayes theorem, *Clostridioides difficile*, diagnosis, polymerase chain reaction, sepsis

## Abstract

Antimicrobial resistance and inappropriate antibiotic use are urgent threats to global health and patient safety. Early antibiotic prescribing often occurs despite uncertain infection status, leading to unnecessary exposure and contributing to resistance. This article advocates for strengthening antimicrobial stewardship by integrating diagnostic stewardship and Bayesian reasoning to more explicitly consider disease probability in clinical decisions. In particular, probability thresholds should be included in clinical guidelines to inform testing decisions that may prompt antibiotic initiation. We provide several clinical examples and highlight the need for clinician education in Bayesian reasoning, underpinned by real-time decision support tools. Anchoring clinical practice in probability-based frameworks promises to refine antibiotic prescribing, enhance patient safety outcomes, and curb the progression of antimicrobial resistance.

## Teaser

Antimicrobial resistance demands more than new antibiotics; it requires smarter prescribing decisions. This article advocates for embedding Bayesian reasoning and explicit probability thresholds into clinical guidelines and decision support tools, offering a practical framework for aligning antibiotic initiation with diagnostic certainty rather than reflex.

In the setting of diagnostic uncertainty, clinicians often err on the side of antibiotic overuse to avoid undertreating bacterial infections especially in high-risk patients, such as immunocompromised patients or those with suspected sepsis. However, this overlooks the equally important harms of unnecessary antimicrobial exposure, for the individual and society, related to adverse events and increased incidence of drug-resistant infections [1].

Clinical guidelines and quality measures built on these guidelines should balance these consequences by defining testing and treating paradigms based on diagnostic certainty. For example, an Infectious Diseases Society of America (IDSA) position paper on the national severe sepsis and septic shock early management bundle (SEP-1) recommends delaying empiric antibiotics when immediate therapy is not clinically urgent, prioritizing diagnostic assessment over reflexive prescribing [2]. In contrast, American Thoracic Society (ATS)/IDSA guidelines recommended antibacterial therapy for patients with community-acquired pneumonia without accounting for the pretest probability of a viral etiology [3]. Diagnostic stewardship, therefore, assumes a critical role in determining which tests to order, when to order, and how to interpret results [4]. The expanding availability of point-of-care tests further justifies the need to consider diagnostic stewardship and Bayesian reasoning in clinical guidelines that adjust therapeutic and diagnostic decision-making to align with stewardship objectives [5].

There are several important terms to define before delving into diagnostic reasoning (Table 1). Prevalence provides a population-level estimate of how common a disease is and often serves as a starting point to estimate the pretest probability of disease, which is the clinician's assessment of the likelihood of disease before testing is performed. Sensitivity (percentage of individuals with the disease who test positive) helps determine how confidently a negative result can rule-out disease. Specificity (percentage without the disease who test negative) indicates how reliably a positive result can rule-in disease. The mnemonics SpIn (high-specificity rules in) and SnOut (high sensitivity rules out) can be helpful reminders. Positive predictive value (PPV) reflects the probability that a positive test truly indicates disease, and negative predictive value (NPV) reflects the probability that a negative test truly indicates the absence of disease; both depend strongly on the underlying prevalence and thus the pretest probability. Likelihood ratios integrate sensitivity and specificity into a single metric that quantifies how much a test result shifts the probability of disease, allowing clinicians to formally update pre-test probability to a post-test probability using Bayesian reasoning. Together, these measures provide a coherent framework for interpreting diagnostic tests and determining whether additional evaluation or treatment is warranted.

**Table 1.** Summary of Statistical Terms and Their Impact on Diagnostic Reasoning

Term	Definition	How It Factors Into Diagnostic Reasoning
Prevalence	The proportion of individuals in a population who have the disease at a given time.	A population-level estimate of disease likelihood; often used as a starting approximation for pretest probability.
Pretest probability	The estimated likelihood that a patient has the disease before any diagnostic test results are known.	Anchors diagnostic reasoning. Derived from prevalence, clinical signs/symptoms, epidemiology, medical history, etc.
Sensitivity	The proportion of patients with the disease who test positive (true positive rate).	High sensitivity helps rule-out disease when negative (SnOut). Informs confidence in negative test results.
Specificity	The proportion of patients without the disease who test negative (true negative rate).	High specificity helps rule-in disease when positive (SpIn). Informs confidence in positive results.
Positive predictive value (PPV)	The probability that a patient with a positive test actually has the disease.	Indicates how likely a positive result can be taken as true. Highly dependent on pretest probability and prevalence.
Negative predictive value (NPV)	The probability that a patient with a negative test truly does not have the disease.	Indicates how likely a negative result can be taken as true. Highly dependent on pretest probability and prevalence.
Likelihood ratios	How many times more likely a test result is in diseased versus non-diseased people.	Convert pretest probability to post-test probability using Bayes’ theorem or a Fagan nomogram; largely independent of prevalence.
Post-test probability	The probability that a patient has (or does not have) the disease after incorporating the test result.	Ultimate output of diagnostic reasoning; reflects how much the test result meaningfully changes disease likelihood.

Diagnostic accuracy is fundamental to effective care and is informed by estimating the patient's pretest probability of disease (ie, initial estimate of disease likelihood prior to testing based on clinical signs/symptoms, epidemiology, medical history, etc.) [6]. Bayes’ theorem offers a quantitative framework for updating disease probability after a test result, integrating pretest probability with test likelihood ratios [6]. Depending on the severity of infection, testing is warranted in cases of intermediate probability and diagnostic uncertainty, rather than at extremes where confirmation offers little additional value or introduces bias. For example, in patients with high pretest probability of disease, positive results only affirm the diagnosis, but false negative results risk undertreatment. Conversely, with low pretest probability, negative results add little diagnostic value while false positive results risk overtreatment. When pretest probability is ambiguous, testing can guide management as positive or negative results rule-in or rule-out disease, respectively.

The term “diagnostic” implies a binary output to indicate the presence or absence of disease. More often, diagnostic tests detect specific biomarkers that increase or decrease the likelihood of disease but are not definitive. An imperfect test may be perceived as unreliable when considered as a standalone diagnostic; however, the same test can be instrumental when interpreted in the context of all available clinical information. Furthermore, diagnostic stewardship should emphasize strategic testing (eg, when to test and how to interpret results), define probability thresholds for infection management in the Bayesian context, and leverage clinical decision support (CDS) to achieve these goals. Application of Bayesian reasoning to specific clinical scenarios illustrates the practicality of this approach, which we highlight with three common infectious disease scenarios.

Bedside clinicians and infection control officers have different goals when testing for *Clostridiodes difficile*: the former to treat active infection, the latter to prevent transmission. While nucleic acid amplification tests (NAATs) are highly sensitive in detecting *C. difficile*, this is not necessarily indicative of *C. difficile* disease. If clinical symptoms suggest a low pretest probability, NAATs could result in false positives and overtreatment. Therefore, guidelines increasingly recommend using NAATs only as confirmatory tests, when pretest probability hovers above 50% based on positive glutamate dehydrogenase or toxin assays [7]. While these guidelines correctly contextualize NAATs in the antimicrobial stewardship (AMS) framework, they undervalue the role of NAATs in infection prevention and control (IPC) where detecting *C. difficile* colonization can trigger IPC interventions [8]. Finally, the shortcomings of the original *C. difficile* infection quality measure, tracked by the National Healthcare Safety Network (NHSN) and incentivized by the Medicare program, demonstrate how unintended consequences of regulatory policies can drive overpenalization and underutilization of testing when testing algorithms are not considered [9]. Diagnostic stewardship guidelines that more clearly define the clinical utility of NAATs can ensure that regulatory policies promote appropriate test-guided treatment and IPC decisions.

For patients with sepsis, minimizing time to appropriate treatment improves health outcomes [10]. High sepsis morbidity and mortality may result in empiric treatment at lower disease certainty, driving the risk of antibiotic overtreatment. This is a prime opportunity for measuring sepsis biomarkers yet they are not routinely used. When used as standalone diagnostic tests, independent of clinical context, these biomarkers fail to appropriately guide sepsis care. However, when interpreted using Bayesian reasoning, these markers can clarify post-test probability and boost diagnostic certainty [11]. New biomarkers should be validated as aids to sepsis diagnosis as part of a structured sequence involving Bayesian updating to determine the need for further testing and to guide management [12]. For example, first-line sepsis tests should have high sensitivity to avoid missing cases of sepsis for patients with a high pretest probability. As new biomarkers enter clinical practice, regularly updated guidelines should evaluate their value in meaningfully shifting post-test probability through Bayesian updating, rather than their ability to independently determine the presence/absence of sepsis. The Surviving Sepsis guidelines exemplify how standard-of-care differs based on definite, probable, and possible sepsis with respect to time-to-antibiotic targets. In contrast, the SEP-1 quality measure illustrates how rigid triage paradigms, such as the mandate for lactate measurements for all suspected sepsis, can interfere with Bayesian-driven diagnostic decision-making [2]. Sepsis triage guidelines based on Bayesian reasoning can steer regulatory policies toward diagnostic excellence without compromising time to appropriate treatment [13].

A final example is bacterial vaginosis (BV). While traditional, high-specificity methods exist to diagnose BV (eg, Amsel criteria and Nugent score), NAATs enable BV detection at higher sensitivity and, in some cases, comparable specificity [14]. Given the high sensitivity of NAATs, current guidelines recommend their use only in symptomatic women to prevent overdiagnosis [15]. Routine BV screening with NAATs among asymptomatic pregnant women for preventing preterm birth is not currently recommended based on mixed results from randomized controlled trials [16]. However, considering the pitfalls of the previous trial designs, the high specificity of some NAATs, and the safety risks associated with missed BV diagnosis in pregnant patients, the clinical utility of specific NAATs should be evaluated for ruling out BV in high-risk asymptomatic pregnant patients. Additionally, the potential benefit of treating asymptomatic BV must be weighed against the potential harms of prenatal antibiotic exposure [17]. Taken together, these studies could justify BV guideline revisions to establish the extent to which pregnancy risks should lower the disease certainty threshold-to-treat and the role of NAATs in this patient population.

Bayesian-driven diagnostic stewardship guidelines can improve patient outcomes by optimizing testing to inform appropriate treatment. Such guidelines could contextualize the clinical utility of testing, supporting market adoption of innovative tests, and align regulatory policies. The performance of new tests must be strategically evaluated within the context of well-established algorithms, which is especially challenging when care pathways lack diagnostic gold standards. It is also imperative that clinicians are equipped with Bayesian reasoning skills as part of medical education, and supported by real-time CDS that ideally integrates into electronic health record systems to facilitate utilization [18, 19]. Clinician biases or inaccuracies in assigning pretest probability and reluctance to use post-test probability to influence decision-making can hamper the impact of Bayesian-informed clinical guidelines.

**Figure 1.**
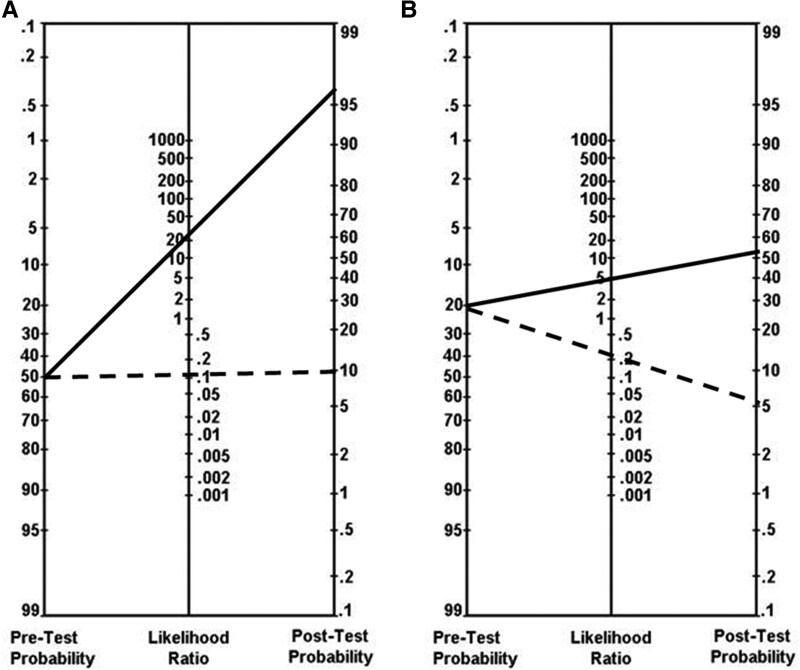
Fagan nomogram. The user inputs the pretest probability and draws a line through the likelihood ratio for a positive (LR+) or negative (LR−) test to derive the post-test probability. In (*A*), a patient with moderate (50%) pretest probability of *C. difficile* infection who undergoes NAAT testing (LR+ 27, LR− 0.11) will have a post-test probability of 96% for a positive test (solid line) but only 10% if negative (dashed line) [21]. In (*B*), a patient with a 20% pretest probability of sepsis undergoes monocyte distribution width (MDW) testing (LR+ 4.7, LR− 0.25) that yields a post-test probability of 54% for an elevated MDW or 6% for a normal MDW [22]. When the pretest probability is imprecise, users could estimate the probability as high, moderate, or low and choose values in those ranges (eg, 90%, 50%, 10%, respectively) to derive estimates. From Fagan [23]. Copyright © 1975 Massachusetts Medical Society. Reprinted with permission from Massachusetts Medical Society.

To reduce biases and inaccuracies in probability quantification, Bayesian principles, pretest probability factors, and test likelihood ratios should be integrated in AI-driven CDS, enhancing the selection and interpretation of diagnostic tests through automatic Bayesian updating of disease probability [20]. Online databases of commonly used tests and their associated likelihood ratios could make this information more universally available. Test developers and manufacturers should also be encouraged to report likelihood ratios in their regulatory submission packages, to make this information freely and readily available. Armed with a pretest probability and test likelihood ratios, interactive tools such as Fagan's nomogram (Figure 1), or digital calculators can quickly determine the impact of test results on disease probability as new clinical information is obtained [23]. A Bayesian approach would help anchor clinical decision-making and test interpretation by quantifying disease probability without increasing the burden on clinicians.

Collectively, these examples highlight the need to develop clinical guidelines that contextualize the value of testing and NAATs in particular based on their impact on post-test probability. Implementing such guidance relies on the development of CDS, integration of Bayesian reasoning in medical education curricula, and finalizing regulatory policies that strengthen AMS. These guidelines should accordingly raise the standard for certainty before antibiotic initiation for all but the most critically ill patients and promote diagnostic excellence through strategic testing.
